# How Are Good Teeth Secured?

**Published:** 1858-09

**Authors:** E. Collins


### HOW ARE GOOD TEETH TO BE SECURED?

BY E. COLLINS.

This subject is one, first in place and importance, compared with the other subjects, selected and presented, by the committee appointed by the American Dental Convention, for discussion at its next annual meeting.

Not expecting to be present and take part in the discussions on this subject, I desire, in as concise manner as possible, to give expression to my views upon it; not that I deem them new to the profession, but because there seems to be a manifest tendency on the part of some to disregard predisposing causes of our physical defects, and to set at nought the importance of forcing home upon the public attention the necessity for their speedy removal, which it is our duty to do, as members of the healing art, as well as to cure disease resulting from those causes.

In order, then, to secure good teeth, we must restore first, to its normal state the health of the physical organization of our race; then, as a natural consequence, the teeth will be found perfect in their organization, and will seldom if ever yield to the action of such foreign substances as were designed by the Creator for man's subsistence.

Congenital diseases which have been, during the last half century, rapidly increasing, and preying upon the vitals of the American people, and thus bringing annually to premature graves thousands of our race, must be counteracted or removed ere we can expect to witness an improvement in the physical character of the teeth, whatever tends to improve the texture of those organs. Hence, the administering to females phosphate of lime, or other tooth elements, during the period of utero-gestation, can accomplish but little until the former end be consummated, and not then unless such substances are craved by the *enciente* mother, for the following reasons: Our physical nature, although we are often untrue to her, is always true to herself, and rarely demands any thing for her nourishment but such as are best adapted to her wants, and within her capacity to elaborate or convert to her own development. Hence, whatever is forced upon her contrary to her demands, either in the above or ordinary circumstances, is repelled as a foreign foe, and she is made thereby to suffer exhaustion of her vital forces, and is more or less manifest in proportion to the offensiveness or inoffensiveness of the substance employed, and thus we increase rather than diminish the evil which we hope to remedy by exciting in those organs, whose offices it is to assimilate and elaborate foreign substances, functional and often structural derangement. If then, females, during utero-gestation, manifest an inordinate craving (as is almost universally the case with those who are uncontaminated by disease), for earthy salts, let them be freely administered; but if not, then it is evident that the due quantity of these salts are received from the ordinary food, and that the system has not the capacity to use an additional quantity, no matter how much such salts may be required to give permanency of texture to the teeth: the teeth of the mother will not always serve for this purpose, for the teeth of the children are often dissimilar to the mother's in the texture, and resemble in form and feature those of the father's, whose teeth may be diametrically opposite to the mother's in character; hence such practice is hap-hazard, and its good results purely imaginary. These, then, are the means to be used for man's redemption, and are the same as those instituted by the Creator for his preservation, viz: strict obedience to the Creator's physical laws. Now, inasmuch as the unenlightened heathen and dumb animals yield obedience to those laws, through what is called instinct, is it not strange that we, endowed as we are with the same nature, instincts with them, do not obey those laws and secure as they do, as a reward for their obedience, strong, healthy, and well formed bodies? At first sight it does seem strange, but when we consider what a powerful influence disease has, in preventing not only the physical, but also the spiritual or intellectual nature of man, which are so materially associated in each others weal or woe, it does not appear so strange. Since judging from the action of the masses of our fellows of the present generation, it does seem that reason and instinct are so extensively perverted that they pressingly demand the help of physical science to restore them again to their normal state. Hence, let these be introduced into all your institutions of learning, a perfect system for training the youths of the present and succeeding generations, in a knowledge of their own physical organization, and the laws which preside over and govern it. And let parents become students of self, and coöperate with teachers in seeing that their children obey those laws, as also the moral laws of the Creator—for those generally go hand in hand—and this alone would ultimately bring about a material decrease in the catalogue of hereditary as well as other diseases, and in restoring our race to their original strength and beauty of bodily conformation ; and thus we would become wiser and better, and in every way fitted in body to ward off the morbific agencies that every where surround us, and defend successfully our republic from the ravages of a foreign foe, and maintain, with honor, down to the latest period of time, those institutions which secure to us, as a nation, the free and inalienable right to life, liberty, and the pursuit of happiness. Again, I would recommend, if it could be rendered consistent with the reading of our national Constitution, that our legislative bodies be petitioned to enact laws prohibiting the marriage relation to take place between parties, both of whom are known to possess any hereditary taint; and only in order to avoid still greater moral and physical depredations than already exists —permiting male invalids to enter into this relation with females who are known to be of healthy parentage, and free themselves from all constitutional diseases.

Now, in conclusion, let me say that until the above or some other means, beyond my power to conceive, be instituted, the teeth of the American people will continue to grow more and more frail and defective, in form and arrangement, until they at last will become very poor apologies for their species. But let the above be put in practical operation and continued, and my word for it the third and fourth generation will not pass until all that I have predicted will be fulfilled, but short of this I will not vouch for: for thus it is written in the divine oracles : "The Lord visiteth the iniquities of the fathers upon the children to the third and fourth generation."

