# Errata

**Published:** 1858-12

**Authors:** 


Erata.—In Dr. Collins' article, in last number of the Register, on "securing good teeth," we are requested to make the following corrections: On page 14, 10 line from top, for "the texture" read their texture. On same page, 13th line from bottom, for "preventing" read perverting, same page 6th line from bottom, for "let there," read let these. On page 15, 15th line from bottom, for "depredations," read degredations.

